# Correction: Grape seed proanthocyanidin extract alleviates high-fat diet induced testicular toxicity in rats

**DOI:** 10.1039/d5ra90018b

**Published:** 2025-02-24

**Authors:** Er Hui Wang, Zeng Li Yu, Yong Jun Bu, Peng Wei Xu, Jin Yan Xi, Hai Yan Liang

**Affiliations:** a Department of Nutrition and Food Hygiene, College of Public Health, Xinxiang Medical University Xinxiang 453003 China weh1985@126.com; b Henan Collaborative Innovation Center of Molecular Diagnosis and Laboratory Medicine, Xinxiang Medical University Xinxiang 453003 China

## Abstract

Correction for ‘Grape seed proanthocyanidin extract alleviates high-fat diet induced testicular toxicity in rats’ by Er Hui Wang *et al.*, *RSC Adv.*, 2019, **9**, 11842–11850, https://doi.org/10.1039/C9RA01017C.

The authors regret an error in Table 1. At the thirteenth week, one recorder recorded the weight on the morning of the experiment day, and another recorder recorded the weight before anesthesia. There was a slight difference between the two pieces of weight data. In order to keep the consistency of academic records, this is being corrected here. The corrected table is shown below. The author apologises for any inconvenience this may cause.

**Table 1:** Mean weekly body weights (g) among groups (mean ± SD, *n* = 10)

Week	Control	HFD	HFD + GSPE (100 mg kg^−1^)	HFD + GSPE (300 mg kg^−1^)
0	129.94 ± 4.77	135.00 ± 5.94	134.60 ± 3.69	128.50 ± 16.61
1	191.30 ± 10.10	215.00 ± 12.84	212.80 ± 7.93	205.70 ± 10.10
2	254.10 ± 15.69	291.70 ± 22.82	288.60 ± 14.24	283.00 ± 14.73
3	300.60 ± 22.66	358.30 ± 32.46	351.70 ± 16.96	343.00 ± 21.53
4	339.60 ± 27.75	407.10 ± 38.52	402.80 ± 21.89	395.70 ± 27.01
5	377.40 ± 31.85	455.10 ± 44.70	441.40 ± 25.26	426.62 ± 42.34
6	406.10 ± 34.26	501.30 ± 52.41	485.00 ± 24.61	472.10 ± 29.30
7	435.10 ± 37.83	531.30 ± 57.58	512.10 ± 30.00	504.20 ± 34.00
8	459.10 ± 39.53	563.50 ± 59.73	543.00 ± 35.90	533.10 ± 28.59
9	481.10 ± 44.50	593.60 ± 58.35	573.80 ± 42.41	564.00 ± 31.16
10	503.20 ± 47.00	625.80 ± 61.95	598.30 ± 48.49	593.80 ± 33.06
11	515.80 ± 55.18	646.30 ± 59.13	617.60 ± 54.10	605.50 ± 33.76
12	526.50 ± 58.57	663.90 ± 53.54	622.00 ± 59.13	612.00 ± 29.66
13	534.00 ± 59.03	695.10 ± 41.58	614.10 ± 59.01	604.00 ± 27.46

An independent expert has viewed the corrected table and has concluded that the data are consistent with the discussions and conclusions presented.

The Royal Society of Chemistry apologises for these errors and any consequent inconvenience to authors and readers.

